# Editorial: Insights in microorganisms in vertebrate digestive systems: 2022

**DOI:** 10.3389/fmicb.2023.1344969

**Published:** 2024-01-05

**Authors:** Yu-Ting Tang, Wei-Qi He

**Affiliations:** Jiangsu Key Laboratory of Neuropsychiatric Diseases and Cambridge-Suda (CAM-SU) Genomic Resource Center, Medical College of Soochow University, Suzhou, Jiangsu, China

**Keywords:** gut microbiota, human diseases, animal husbandry, microbial imbalances, physiological impact

The entire microbiota of the digestive tract, spanning from the oral cavity to the rectum, is established through interactions with the external environment, a process initiated during the fetal stage (Martino et al., 2022). Microorganisms intricately influence host physiological activities (Westermann and Vogel, 2021), engaging with the nervous system, immune system, and various organs (Morais et al., 2021; Tilg et al., 2022). Imbalances in the microecology impact diverse aspects of the host's cognition, emotions, diet, and metabolism (Fan and Pedersen, 2021). Deyaert et al. established an *in vitro* dynamic ileal microbiota model to investigate bacterial activity. Additionally, Pan et al. highlighted the regulatory role of flavonoids in shaping the human gut microbiota structure. We underscore that an understanding of microbial ecology can unveil the host's health status. This Research Topic aims to provide a comprehensive overview of cutting-edge studies on the symbiotic relationship between microorganisms and digestive system. This aligns with the summaries provided by Liang et al., Kasarello et al., and White et al. contributing insights and addressing current challenges in the exploration of microorganism interactions across multiple systems.

The gut microbiota actively participates in the human physiological and pathological activities (Shalon et al., 2023). Its potential as prognosis indicators or treatment targets has been deeply explored. Hu et al. illustrated the microbial landscape of the gallbladder in patients with gallstones, revealing significant alterations in bacterial taxonomic composition and strengthened correlation between bacterial and fungal communities in bile, potentially linked to gallstone formation. Xiang et al. connected the saliva microbiota changes with the renal function recovery in renal transplant patients during the perioperative period, suggesting them as biomarkers for postoperative recovery. Jiao et al. reported that a rising abundance of *Actinobacteria* and *Bifidobacterium* in the intestine following salidroside treatment, correlated with downregulation of *Tomm7* (*translocase of outer mitochondrial membrane 7*) in the hippocampus, ameliorating memory impairment after long-term ethanol intake in rats. Zhao M. et al. proposed that an increase in *Shigella* and a decrease in *Prevotella* and *Bacteroides* may contribute to the occurrence and development of myasthenia gravis. The imbalance of the microbial community can be adjusted by Modified Buzhong Yiqi Decoction treatment, further enhancing host immune function.

Beyond its implications for human diseases, the gut microbiota holds the potential for developments in animal husbandry. Ji et al. proposed that organic Mn supplementation in the diet had more advantages than the sulfate forms in weaning calves, maintaining a more stable microbial community. Zhao W. et al. demonstrated that a diet supplemented with glucose oxidase may strengthen the immunologic barrier and maintain a healthy intestinal microecology. A deeper understanding of the microbiota community aids in optimizing gut function and improving feeding efficiency. Zhang et al. documented dynamic changes in fecal microbiota in donkey foals during weaning, while He et al. manipulated gut microbial and metabolic profiles through hybridization in Tunchang pigs. Regarding microbial structure, composition, and functional capacity as crucial assessment, Li et al. disclosed that house ammonia exposure in rabbits may impact on local immune responses and inflammatory processes. Liu et al. disclosed a higher prevalence of multidrug-resistant *Proteus mirabilis* in domestic dogs, along with corresponding antibiotic resistance genes. This finding emphasizes the importance of prudent antibiotic management by veterinarians. The study of vertebrate microbiota offers an opportunity to comprehensively monitor the health status of livestock, ensuring the high-quality of husbandry products (Wen et al., 2021).

It is inspiring that our Research Topic has garnered attention from researchers worldwide, including those from the USA, Poland, Belgium, the Netherlands, and China. We extend our gratitude to all authors who contributed their original work to this Research Topic and the reviewers for their invaluable comments. We also express our sincere thanks to the editorial office of Frontiers in Microbiology for their excellent support and for providing us with the opportunity to successfully host this hot topic issue.

## Author contributions

Y-TT: Writing—original draft, Writing—review & editing. W-QH: Funding acquisition, Writing—original draft, Writing—review & editing.
